# Comparison of cardiac DTI parameters between systole and diastole

**DOI:** 10.1186/1532-429X-16-S1-P39

**Published:** 2014-01-16

**Authors:** Laura-Ann McGill, Pedro Ferreira, Andrew D Scott, Sonia Nielles-Vallespin, Ranil Silva, Philip J Kilner, David Firmin, Dudley J Pennell

**Affiliations:** 1 Royal Brompton, Sydney Street, London, UK; 2 National Heart, Lung and Blood Institute., National Institute of Health, Bethesda, Maryland, USA

## Background

*In vivo *Cardiac Diffusion Tensor Imaging (cDTI) offers the potential to calculate the mean intravoxel myocyte helical angle (HA) and assess the degree of average myocyte organisation with fractional anisotropy (FA). However it is not known how these parameters compare between different phases of the cardiac cycle.

## Methods

We recruited 46 healthy volunteers for cDTI[1] at 3T. Three short axis mid-ventricular slices were acquired with multiple breath holds during both the end systolic and end diastolic pauses. Data was post-processed with a platform, developed in-house, to create FA, HA and mean diffusivity (MD) maps.

## Results

Two of the original 46 volunteers were excluded due to ECG irregularities. Data from one further volunteer was incomplete and therefore excluded from the final analysis. Results from the remaining 43 volunteers are in table 1. Global FA was higher in diastole than systole (0.56 v 0.47; p < 0.001). The global endocardial HA was significantly more right-handed in systole than diastole (34° v 25°; p < 0.001). The global mesocardial HA was circumferentially orientated and similar in both diastole and systole (-3° v -2°; p = 0.42). The global epicardial HA was slightly more left-handed in systole than diastole (-35° v -30°; p < 0.001). Global MD was higher in diastole than systole (1.11 v 0.93 × 10^-3^mm^2^/s; p < 0.001).

**Table 1:** 

**Baseline Characteristics **	N = 43
Age: yrs (range)	45 (24-74)

Male subjects	26 (60%)

BSA: m^2^	1.88 ± 0.20

BMI: kg/m^2^	24.5 ± 2.98

LVEDVi: ml/m	77.5 ± 14.1

LVMi: g/m^2^	63.7 ± 17.1

LVEF: %	68 ± 6

**DTI Parameters **	

*Fractional anisotropy	

Diastole	0.56 ± 0.04

Systole	0.47 ± 0.05

*Endocardial HA global: °	

Diastole	25 ± 5

Systole	34 ± 5

**Mesocardial HA global**: °	

Diastole	-3 ± 4

Systole	-2 ± 3

*Epicardial HA global: °	

Diastole	-30 ± 6

Systole	-35 ± 5

*MD global: ×10 ^-3^mm^2^/s	

Diastole	1.11 ± 0.13

Systole	0.93 ± 0.14

Mean ± SD, *p < 0.001

## Conclusions

The MD measurement with this sequence is expected to be independent of the phase at which it is sampled. Our finding of a significant difference between systole and diastole may therefore be as a consequence of strain effects which have been reported to distort the diffusion tensor when acquired out with the cardiac 'sweet spot'[2]. The finding of a higher FA in end diastole must therefore be interpreted with caution, however one explanation is that myocytes in diastole are more anisotropic than contracted systolic fibres. Our finding that the mesocardial HA is circumferentially orientated in both phases is consistent with ex vivo studies[3]. The systolic increase in proportions of righthand and lefthand sloping HAs in the endocardium and epicardium, respectively, may in part be methodological being potentially attributable less inclusive segmentation of these layers when the wall is thin at end diastole. Our current work is been directed towards acquiring 3D strain data to establish the impact of strain, and other technical factors, on the diffusion tensor at different phases.

## Funding

NIHR cardiovascular BRU of Royal Brompton Hospital & Imperial College.
